# ELAVL1 is transcriptionally activated by FOXC1 and promotes ferroptosis in myocardial ischemia/reperfusion injury by regulating autophagy

**DOI:** 10.1186/s10020-021-00271-w

**Published:** 2021-02-10

**Authors:** Hui-Yong Chen, Ze-Zhou Xiao, Xiao Ling, Rong-Ning Xu, Peng Zhu, Shao-Yi Zheng

**Affiliations:** 1 Department of Cardiovascular Surgery, Nanfang Hospital, Southern Medical University, No.1838 North Guangzhou Avenue, Baiyun District, Guangzhou, 510515 Guangdong People’s Republic of China; 2 Department of Thoracic Surgery, Yuebei People’s Hospital, Shantou University, Shaoguan, 512026 Guangdong People’s Republic of China

**Keywords:** Myocardial ischemia reperfusion, Ferroptosis, Autophagy, ELAVL1, FOXC1

## Abstract

**Aims:**

Myocardial ischemia is the most common form of cardiovascular disease and the leading cause of morbidity and mortality. Understanding the mechanisms is very crucial for the development of effective therapy. Therefore, this study aimed to investigate the functional roles and mechanisms by which ELAVL1 regulates myocardial ischemia and reperfusion (I/R) injury.

**Methods:**

Mouse myocardial I/R model and cultured myocardial cells exposed to hypoxia/reperfusion (H/R) were used in this study. Features of ferroptosis were evidenced by LDH activity, GPx4 activity, cellular iron, ROS, LPO, and GSH levels. The expression levels of autophagy markers (Beclin-1, p62, LC3), ELAVL1 and FOXC1 were measured by qRT-PCR, immunostaining and western blot. RIP assay, biotin-pull down, ChIP and dual luciferase activity assay were employed to examine the interactions of ELAVL1/Beclin-1 mRNA and FOXC1/ELAVL1 promoter. CCK-8 assay was used to examine viability of cells. TTC staining was performed to assess the myocardial I/R injury.

**Results:**

Myocardial I/R surgery induced ferroptosis and up-regulated ELAVL1 level. Knockdown of ELAVL1 decreased ferroptosis and ameliorated I/R injury. Si-ELAVL1 repressed autophagy and inhibition of autophagy by inhibitor suppressed ferroptosis and I/R injury in myocardial cells. Increase of autophagy could reverse the effects of ELAVL1 knockdown on ferroptosis and I/R injury. ELAVL1 directly bound with and stabilized Beclin-1 mRNA. Furthermore, FOXC1 bound to ELAVL1 promoter region and activated its transcription upon H/R exposure.

**Conclusion:**

FOXC1 transcriptionally activated ELAVL1 may promote ferroptosis during myocardial I/R by modulating autophagy, leading to myocardial injury. Inhibition of ELAVL1-mediated autophagic ferroptosis would be a new viewpoint in the treatment of myocardial I/R injury.

## Introduction

Myocardial ischemia occurs when the blood flow to the heart is clogged or reduced, resulting in impaired oxygen and nutrient supply to heart tissues like cardiac muscle and subsequent heart dysfunction (Frank 2012). It is the most common form of coronary heart disease, and the leading cause to morbidity and mortality worldwide (Aguero 2015). Proper and timely restoration of the blood flow through anti-thrombolytic drugs or mechanical interventions is the primary treatment (Lu et al. 2015). However, those treatments are restricted by a narrow time window and side effects. Moreover, the reperfusion process would induce a secondary injury. Many mechanisms have been proposed to mediate the reperfusion-induced injury, including inflammation and altered metabolism (Frank 2012; Hausenloy and Yellon 2013; Turer and Hill 2010). Nevertheless, due to its complexity, a clear picture is still lacking. Given the current situation, it is very necessary to understand the molecular mechanism of myocardial ischemia/reperfusion (I/R) injury so that effective therapeutic strategies could be developed.

Ferroptosis is a novel form of programmed cell death featured by a great amount of cellular iron and lipid peroxides (Li 2020). The process is iron-dependent and involves an enhanced accumulation of lipid reactive oxygen species (ROS) and a compromised glutathione (GSH)-dependent antioxidant system (Xie 2016). Further, previous studies have shown that ferroptosis is connected to autophagy, a conserved process that helps maintain cell homeostasis by degrading dysfunctional organelles and proteins in the autophagosomes, and to some extent ferroptosis is dependent on autophagy (Zhou 2019a, 2019b). Autophagy is largely involved in and significantly contributes to the myocardial I/R injury (Wu et al. 2019). In addition, emerging evidence implicates a critical role of ferroptosis in myocardial I/R injury as iron deposition and overproduction of ROS have been observed during diabetic myocardial damage (Li et al. 2020), although the exact role of ferroptosis in myocardial I/R injury is not well understood.

ELAVL1 (embryonic lethal-abnormal vision like protein 1) is an AU-rich element (ARE) and U-rich element RNA binding protein (RBP) that regulates gene expression by stabilizing message RNAs (mRNAs) (Simone and Keene 2013). By binding with the 3′ UTR of target mRNAs, ELAVL1 promotes the expression of many genes, such as VEGF-A, an angiogenic factor, and TNFα (Levy et al. 1998; Wang 2013). As a result, ELAVL1 plays a crucial role in many cellular processes, including angiogenesis, apoptosis, and inflammation (Wang 2013; Srikantan and Gorospe 2012). Previous studies have reported an elevation in ELAVL1 level in human diabetic heart and implicated its role in cardiac pyroptosis, as well as ferroptosis in liver fibrosis (Bruin et al. 2017; Zhang 2018). Further, knockdown of ELAVL1 attenuates inflammatory responses during myocardial infarction (Krishnamurthy 2010). We, therefore, hypothesized that ELAVL1 might contribute to myocardial I/R injury, despite that the relevant studies of the role of ELAVL1 in myocardia I/R are limited.

Forkhead box C1 (FOXC1) is a transcription factor that belongs to the FOX family featured by the conserved “fork-head” or “winged-helix” DNA-binding domain (Hannenhalli and Kaestner 2009). It has diverse functions like cell growth, metabolism, and survival (Hannenhalli and Kaestner 2009). Some previous studies indicate that FOXC1 is a hypoxia-activated transcription factor to promote cancer cell growth (Lin 2017). In addition, some literature suggests that FOXC1 could be induced during myocardial ischemia (Zhang 2019). The underlying mechanisms of FOXC1 activation during myocardial I/R are elusive. Our preliminary bioinformatic analysis suggested that FOXC1 may bind ELAVL1 promoter. We thus hypothesized that ELAVL1 might be a downstream effector of FOXC1 in myocardial I/R.

In the present study, we sought to investigate the function of ferroptosis in myocardial I/R injury by focusing on FOXC1/ELAVL1 interaction. We confirmed that FOXC1 transcriptionally activated ELAVL1 greatly contributes to myocardial I/R injury via increasing autophagic ferroptosis. Our study provides insights into the mechanisms of myocardial I/R and avenues for the development of future therapy.

## Materials and methods

### Mouse myocardial ischemia/reperfusion (I/R) model

All animal experiments have been approved by the Committee on Animal Care and Use of Nanfang Hospital, Southern Medical University and were performed according to the guidance. Healthy male C57BL/6 J mice (8 weeks) were purchased from Shanghai SLAC laboratory Animal Co., Ltd. (Shanghai, China) and kept in the standard animal facility. To induce myocardial I/R injury, mice were anesthetized by ketamine and xylazine (250 and 10 mg/kg, respectively) first and maintained on 3% isoflurane. An abdominal incision was made around the fourth intercostal space and the left anterior descending coronary artery (LAD) was exposed. LAD was ligated with the silk suture for 1 h followed by 4 h of perfusion. During the perfusion, the incision was closed. For the sham group, same surgery procedures were performed without any ligation. For inhibition of ELAVL1 or overexpression of Beclin-1 in mice, lentivirus vectors (1 × 10^8^ titers) obtained from GeneChem (Shanghai, China) were used for left ventricular cavity injection. After 7 days of lentivirus infection, mice were subjected to myocardial I/R surgery.

### Cell culture and treatments

Human cardiomyocytes (HCM cells) were obtained from American Type Culture Collection (ATCC, Manassas, VA, USA) and used for the experiments. Cells were cultured in Dulbecco's modified Eagle's medium (DMEM; Sigma-Aldrich, USA) supplemented with 10% (v/v) fetal bovine serum (FBS; GIBCO, USA) and 2 mM glutamine (GIBCO), and maintained at 37 °C in a humidified incubator containing 5% CO_2._ For overexpression of Beclin-1, Beclin-1 cDNA was cloned into the overexpression construct (pcDNA3.1). Small interfering RNA of ELAVL1 (si-ELAVL1) and FOXC1 (si-FOXC1), negative control (NC) were synthesized and purchased from GeneChem. The transfection was performed using Lipofectamine 3000 (Invitrogen, Missouri, USA) according to the manufacturer’s guidance. Briefly, cells were grown up to 60–80% confluence and then added with 1 μg plasmid together with 1 μL Lipofectamin 3000. To mimic the myocardial I/R injury in vitro, HCM cells were subjected to hypoxia followed by reoxygenation (H/R). Briefly, cells were placed in an anaerobic chamber (95% N_2_ and 5% CO_2_) at 37 °C for 2 h. Subsequently, the cells were put back in the normal culture condition with 95% air and 5% CO_2_ for another 24 h. Control cells were cultured under normal culture conditions.

### Cell counting kit-8 (CCK-8) assay

Cell proliferation was measured using the standard CCK-8 kit according to the manufacturer’s instruction. Cells were plated in the 96-well plates and cultured in the incubator. 10 µL CCK-8 solution was added to each well and incubated at 37 °C for 2 h. The absorbance at 450 nm was analyzed with the standard microplate reader.

### Iron level detection assay

Intracellular iron level was determined with the standard Iron assay kit (Abcam, USA) according to the manufacturer’s protocol. Cultured or transfected cells following treatments were lysed with RIPA lysis buffer supplemented with protease inhibitor. Protein concentration in extracted lysate was quantified with the pierce BCA protein Assay (Thermo Fisher Scientific, USA). Iron levels in the extracted lysate was similarly quantified with the Iron assay kit. Iron level in protein lysate was calculated. The iron level detection of tissues was the same as above.

### Cellular ROS detection assay

ROS level was determined with the standard ROS detection kit (ab113851; Abcam, USA) based on the manufacturer’s protocol. The HCM cells were plated into 96-well culture plates at a density of 2.5 × 10^4^ cells/well. Cultured or transfected cells following treatments were incubated with the ROS working solution at 37 °C for 45 min in the dark and the fluorescence intensity was analyzed.

### Cellular glutathione (GSH) level detection

Intracellular GSH level was measured with the standard GSH detection assay kit (Nanjing Jiancheng Bioengineering Institute, China) according to the manufacturer’s instruction. The HCM cells (1 × 10^5^/well) were seeded into 24-well plates and cultured overnight. Then, treated cells were collected and broken, supernatants were well mixed with precipitant, buffer and developer. After standing for 5 min, the absorbance at 405 nm was measured using a microplate reader. The levels of GSH in left ventricular myocardium were measured following the described protocol above.

### Lactate dehydrogenase (LDH) activity assay

The activity of LDH was assessed with the standard LDH activity assay kit (Nanjing Jiancheng). Cell culture supernatants or serum (20 µL) were collected and added to master reaction mix according to the manufacturer’s instruction. Five minutes after reaction, the absorbance at 450 nm was analyzed with the reader.

### Glutathione peroxidase 4 (GPx4) activity detection

The lysates of myocardium were collected for GPx4 activity measurement by using an enzyme-linked immunosorbent assay (ELISA) kit (Shanghai Jianglai Biotechnology Co., Ltd., China). In short, equal amount (50 µL) of standards and samples were added into corresponding wells and incubated for one hour at 37 °C followed by addition of 50 μL reaction substrate for 15 min incubation in dark at 37 °C. After adding 50 μL of termination solution into each well, the absorbance was analyzed at the wavelength of 450 nm with the microplate reader immediately.

### Lipid hydroperoxide (LPO) assay

LPO level in cells or lysates of myocardium was determined with the standard kit (Nanjing Jiancheng). Briefly, lipid peroxide was incubated with chromogenic reagents under the condition of 45 °C for 60 min followed by centrifugation for 10 min. The absorbance of supernatants at 586 nm were measured with the microplate reader.

### RNA extraction and qRT-PCR

Trizol reagent (Invitrogen, Missouri, USA) was used to isolate total RNAs from cultured cells or left ventricular myocardium tissues according to the manufacturer's instructions. DNaseI was included into the lysis buffer to avoid the contamination of DNA. 1 μg total RNA of each sample was used for reverse transcription and then amplified by PCR with standard kits (Invitrogen, Missouri, USA). Relative expression levels of mRNA were normalized to GAPDH as internal controls. The following primers were used: ELAVL1: 5′-CGCAGAGATTCAGGTTCTCC-3′ (forward), 5′-CCAAACCCTTTGCACTTGTT-3′ (reverse); GPx4: 5′-GTGGAACTTCACCAAGTTTGGAC-3′ (forward), 5′-GGGCAGGTCCTTCTCTATCAC-3′ (reverse); Beclin-1: 5′-GAGAACCTCAGCCGAAGACT-3′ (forward), 5′-CCTCTAGTGCCAGCTCCTTT-3′ (reverse); p62: 5′-GTACCAGGACAGCGAGAGGAA-3′ (forward), 5′-CCCATGTTGCACGCCAAAC-3′ (reverse); LC3: 5′-GAAGTTCAGCCACCTGCCAC-3′ (forward), 5′-TCTGAGGTGGAGGGTCAGTC-3′ (reverse); FOXC1: 5′-TAGCTACATCGCGCTCATCA-3′ (forward), 5′-ACCTTGACGAAGCACTCGTT-3′ (reverse); GAPDH: 5′-CCAGGTGGTCTCCTCTGA-3′ (forward), 5′-GCTGTAGCCAAATCGTTGT-3′ (reverse); ELAVL1 (mouse): 5′- ACTGCAGGGATGACATTGGG-3′ (forward), 5′-CCAAGCTGTGTCCTGCTACT-3′ (reverse); Beclin-1 (mouse): 5′-CAGTGTTCCTGTGGAGTGGA-3′ (forward), 5′-TGCACACAGTCCAGAAAAGC-3′ (reverse); GAPDH (mouse): 5′- AGCCCAAGATGCCCTTCAGT-3′ (forward), 5′-CCGTGTTCCTACCCCCAATG-3′ (reverse).

### Western blotting

RIPA lysis buffer (ThermoFisher, MI, USA) was utilized to extract proteins from left ventricular myocardium tissues or cells according to standard protocol. Protein concentration of each sample was measured by using Pierce™ BCA Protein Assay Kit (ThermoFisher, MI, USA). Equal amounts of protein were loaded into SDS–polyacrylamide gels and separated through electrophoresis. Later the proteins were transferred from the gels to PVDF membranes (Sigma-Aldrich, USA). The membranes were blocked with 3% BSA for half an hour at room temperature and then incubated with primary antibodies overnight at 4 °C. On the next day the membranes were washed with TBST 3 times before incubation with specific secondary antibodies for 1 h at room temperature. Signals were detected by using the standard ECL kit. Primary antibodies used in the study were: Anti-ELAVL1 (1:1000; Abcam, USA); Anti-GPx4 (1:5000; Abcam); Anti-ferritin heavy chain (FTH1, 1:1000; Abcam); Anti-Beclin-1 (1:1000; Santa Cruz, USA); Anti-p62 (1:1000; Abcam); Anti-LC3 (1:3000; Abcam); Anti-FOXC1 (1:1000; Abcam); Anti-β-actin (1: 1000; Santa Cruz).

### Chromatin immunoprecipitation (ChIP) Assay

ChIP was performed using the commercial ChIP kit (Cell signaling technology, USA) according to the manufacturer’s protocol. For each chromatin immunoprecipitation, 5 μg of anti-FOXC1 and 1 μL of normal rabbit IgG were used. IgG antibody was included as a negative control. After immunoprecipitation, chromosomal DNA was purified. ELAVL1 promoter region was detected by using qRT-PCR.

### RNA Immunoprecipitation (RIP) assay

Transfected cells were lysed in lysis buffer (50 mM Tris–HCl, 150 mM NaCl, 2 mM EDTA, 1% NP-40, 0.5% sodium deoxycholate) containing RNase inhibitors and protease inhibitors (Thermo Scientific, Waltham, MA, USA). The extracted protein was incubated with relevant antibodies (anti-ELAVL1 and IgG as control) (Abcam, USA) overnight at 4 °C and then pulled down with protein G Sepharose 4 Fast Flow suspension (GE Amersham, Little Chalfont, UK). The beads were digested with proteinase K (Sangon, Shanghai, China) for 1 h followed by RNA purification with Trizol reagent (Invitrogen, Missouri, USA). qRT-PCR was performed to examine the RNA yield. The primers were listed in the qRT-PCR section.

### Biotin pull-down assay

The biotinylated transcripts (3′UTR of Beclin-1 mRNA, 5′UTR, or the coding region) were prepared using the MEGshortscript™ T7 kit based on the manufacturer’s protocol and then incubated with cell lysates from transfected myocardial cells at 4 °C for 1 h followed by mixture with streptavidin-coupled dynabeads (Invitrogen, Shanghai, China) at 4 °C for another 3 h. The beads were then washed with TENT (10 mM Tris–HCl [pH 8.0], 1 mM EDTA [pH 8.0], 250 mM NaCl, 0.5% Triton X-100) buffer 3 times and eluted with 1 × laemmli SDS sample buffer. The elution was heated for 5 min at 95 °C and proceeded to western blotting.

### RNA stability

For RNA stability assay, cells with treated with actinomycin D (5 µg/mL) for 0, 2, 4 h followed by RNA extraction using TRIzol reagent. Then, qRT-PCR was performed to assess mRNA remaining.

### Immunostaining

Cells were fixed in 4% paraformaldehyde (PFA) at room temperature for 10–15 min and permeabilized with 0.1% Triton X-100 in PBS for half an hour at room temperature. Then cells were blocked with 1% BSA in PBS for 1 h at room temperature followed by incubation with primary antibody anti-LC3 (1: 500; Thermo Fisher Scientific, USA) at 4 °C overnight. Cells were then washed with PBS and incubated with secondary antibody conjugated with TRITC conjugated secondary antibody for 2 h at room temperature. DAPI was used to stain nucleus. Images were acquired with standard microscope.

### Dual luciferase reporter assay

Fragments of the cDNAs containing the promoter region of ELAVL1 were amplified by PCR and were cloned into the Sall and bamHl restriction sites (Promega, USA) of the luciferase report gene of pmirGLO. Myocardial cells were cultured in 24-well culture plates for 12 h and then recombinant plasmids were co-transfected together with FOXC1 or empty vector into cells. The co-transfected cells were lysed using the Reporter Lysis Buffer. The luciferase activity of each sample was measured using the Dual-Luciferase Reporter Assay System (Promega, WI, USA).

### Infarct size measurement

The infarct size was determined by 2,3,5-triphenyltetrazolium chloride (TTC) staining. Firstly, 1 mL 1% Evans blue dye in 0.9% NaCl was injected via the aorta to visualize the area at risk (AAR), and the hearts were immediately stored at -20 °C for 1 h followed by coronally sectioning at 2 mm. The sections were then stained with 1% TTC for 10 min at 37 °C. Images were analyzed with ImageJ and live area, infarct area, AAR were measured by two blinded, independent operators. Lastly, infarct size was prestented as the percentage of AAR.

### Statistical analysis

All experiments were performed with at least three biological replicates and the data were presented as Mean ± SD. All statistical analyses were analyzed in GraphPad Prism 7. Statistical significance was determined by unpaired Student *t* test or one-way ANOVA followed by Tukey’s post test as indicated in figure legends. *P* < 0.05 was considered as statistically significant.

## Results

### Myocardial I/R induces ferroptosis and increases ELAVL1 expression level

**Fig. 1:**
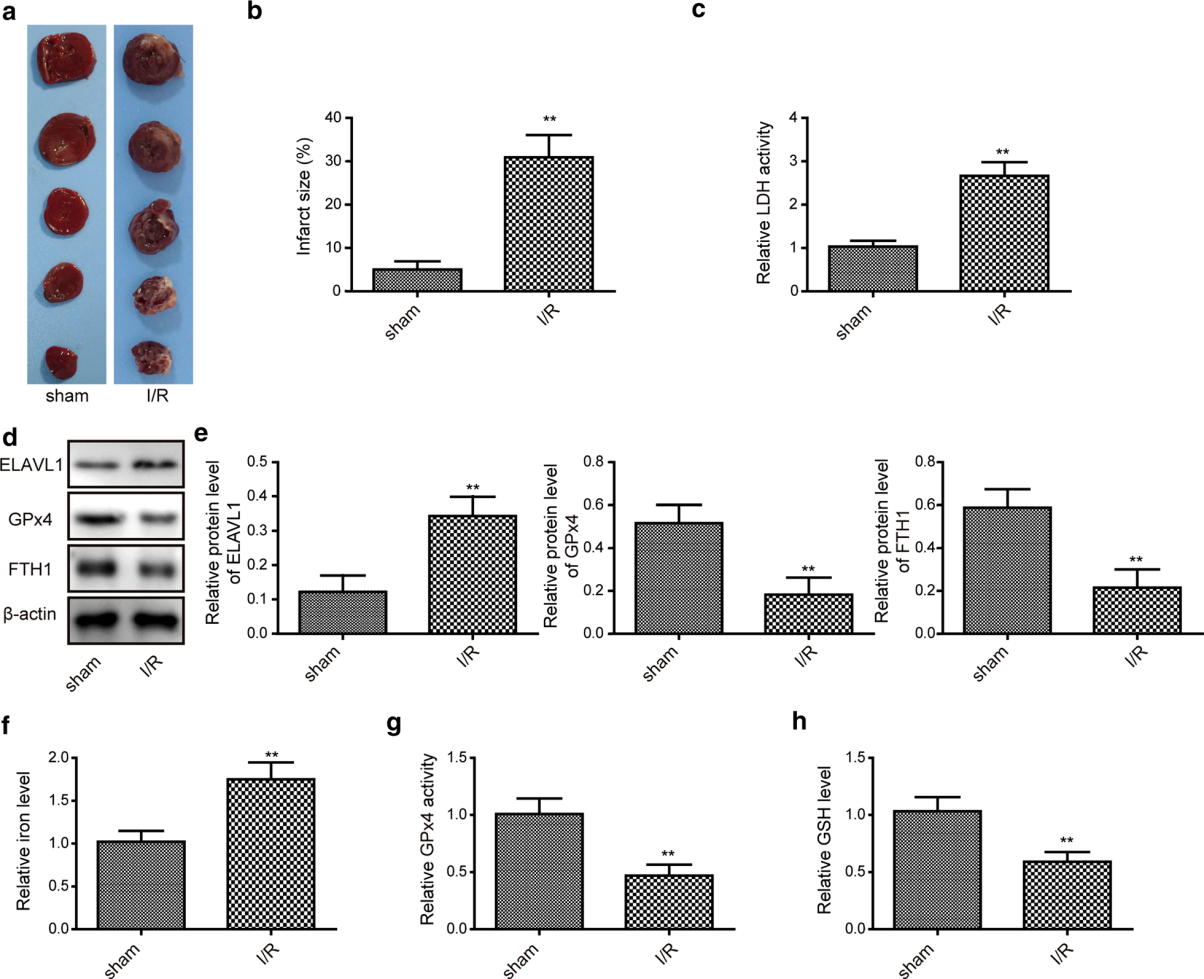
Myocardial I/R induces ferroptosis and increases ELAVL1 expression level. Mice were subjected to myocardial I/R or sham surgery. **a** Representative TTC staining images of myocardial sections from sham-surgery mice and I/R surgery mice. **b** Quantifications of infarct size in each group. **c** Relative LDH activity in sham-surgery group and I/R group. **d**, **e** Relative protein levels of ELAVL1, GPx4, and FTH1 in sham-surgery group and I/R group. **f** Relative cellular iron levels in sham-surgery group and I/R group. **g** Relative GPx4 activity in sham-surgery group and I/R group. **h** Relative cellular GSH level in sham-surgery group and I/R group. The results were presented as the mean ± SD. n = 5; **P < 0.01

To study the role of ferroptosis and ELAVL1 in myocardial I/R injury, we established the mouse myocardial I/R model. As shown in Fig. 1a with the TTC staining, myocardial I/R surgery caused a remarkable infarct area in the heart compared to the sham surgery (Fig. 1a, b). Then, we measured several parameters of ferroptosis, including LDH and GPx4 activity, FTH1, iron, and GSH levels. The results showed that I/R surgery significantly up-regulated LDH activity and cellular iron levels but greatly decreased GPx4 activity, FTH1 and GSH levels (Fig. 1c–h), indicating that ferroptosis occurs during myocardial I/R. Further, we found that ELAVL1 was remarkably elevated following I/R injury (Fig. 1d). These data show that ferroptosis is involved in myocardial I/R injury and ELAVL1 may play a pivotal role during this process.

### Knockdown of ELAVL1 suppresses ferroptosis and H/R injury

**Fig. 2:**
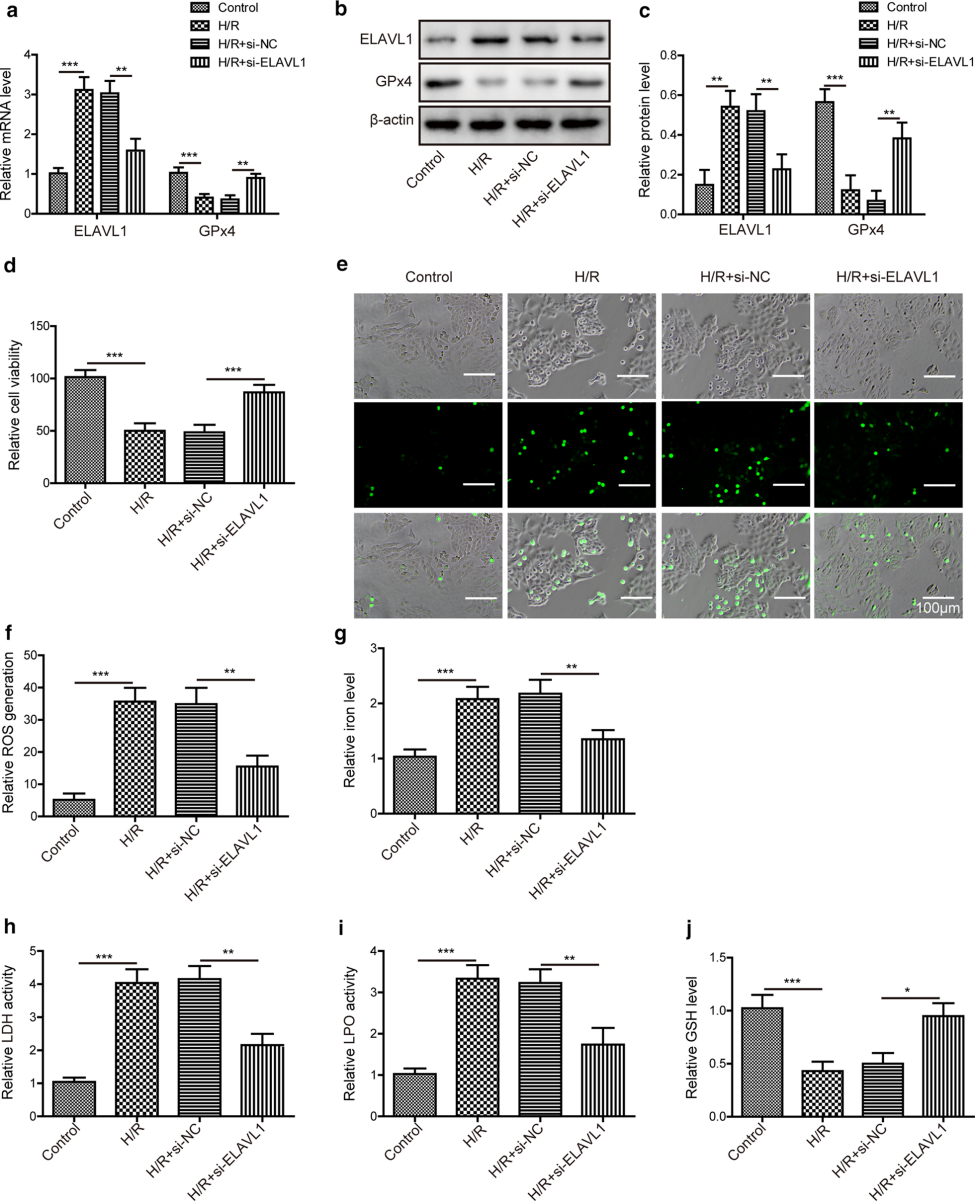
Knockdown of ELAVL1 suppresses ferroptosis and H/R injury. Myocardial cells were transfected with si-ELAVL1 or si-NC and then subjected to H/R. **a** Relative mRNA levels of ELAVL1 and GPx4 in transfected myocardial cells following H/R exposure. **b**, **c** Relative protein levels of ELAVL1 and GPx4 in transfected myocardial cells following H/R exposure. **d** CCK8 assay to assess viability of transfected cells upon H/R exposure. **e**, **f** Relative cellular ROS levels were measured in each group of cells. Relative cellular iron level (**g**), LDH activity (**h**), LPO level (**i**) and GSH level (**j**) in each group were detected respectively. The results were presented as the mean ± SD. n = 3; *P < 0.05, **P < 0.01, ***P < 0.001

To test the role of ELAVL1 in myocardial I/R injury, we decreased ELAVL1 level by siRNA and measured its ensuring effects. We mimicked the myocardial I/R injury in vitro by exposing the cultured myocardial cells to hypoxia followed by reoxygenation. Consistently, H/R exposure increased ELAVL1 mRNA and protein levels but decreased GPx4 levels in myocardial cells (Fig. 2a–c). Transfection of cells with si-ELAVL1 suppressed the H/R induced upregulation of ELAVL1, as expected (Fig. 2a–c). Also, knockdown of ELAVL1 recovered the mRNA and protein levels of GPx4 (Fig. 2a–c). With CCK-8 assay, we showed that H/R substantially decreased the viability of myocardial cells while knockdown of ELAVL1 partially restored (Fig. 2d). In addition, H/R exposure enhanced the levels of intracellular ROS, iron, LPO levels, and LDH activity but decreased cellular GSH level (Fig. 2e–j). However, knockdown of ELAVL1 restored those levels caused by H/R (Fig. 2e–j ). Taken together, these results make clear that knockdown of ELAVL1 suppresses H/R induced ferroptosis and thus ameliorates cell injury.

### ELAVL1 regulates H/R induced autophagy

**Fig. 3:**
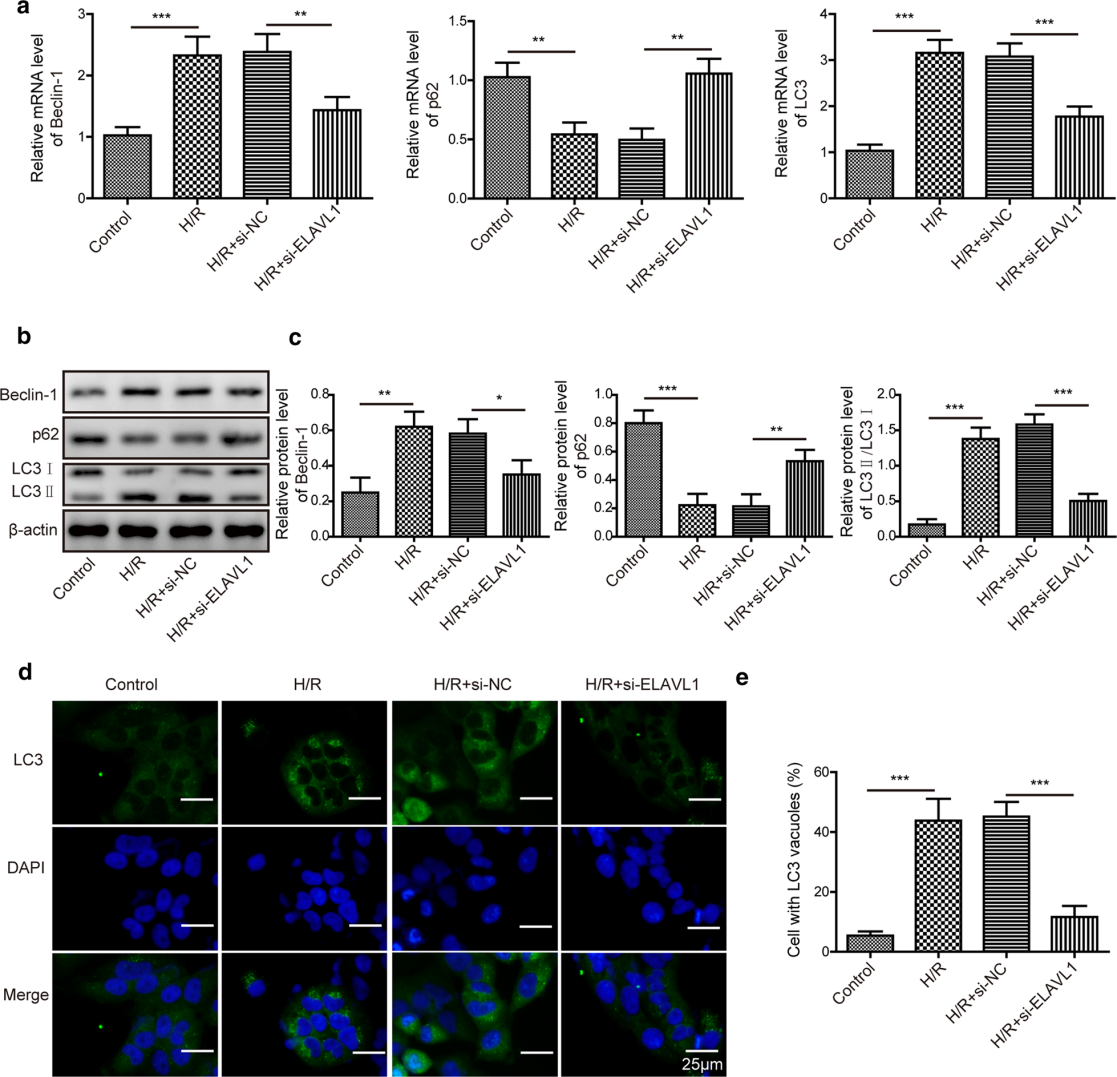
ELAVL1 regulates H/R induced autophagy. Myocardial cells were transfected with si-ELAVL1 or si-NC and then subjected to H/R. **a** Relative mRNA levels of Beclin-1, p62 and LC3 were detected by qRT-PCR. **b**, **c** Western blotting was allowed to assess relative protein levels of Beclin-1, p62 and LC3 in transfected cells following H/R. **d**, **e** Immunostaining analysis of cellular LC3 levels in transfected cells following H/R. The results were presented as the mean ± SD. n = 3; *P < 0.05, **P < 0.01, ***P < 0.001

Ferroptosis is closely related to autophagy. To study how ELAVL1 regulated ferroptosis, we examined the level of autophagy during H/R injury. H/R exposure drastically increased the mRNA and protein levels of Beclin-1 and LC3 but diminished p62 levels (Fig. 3a–c). Interestingly, Knockdown of ELAVL1 in myocardial cells reverse those changes induced by H/R (Fig. 3a–c). Moreover, with immunostaining of LC3 signal, we found H/R exposure greatly raised the intracellular level of LC3 while knockdown of ELAVL1 suppressed that rise (Fig. 3d, e). Therefore, we conclude that knockdown of ELAVL1 inhibits H/R induced autophagy, which might be the mechanism of its anti-ferroptosis effect.

### Inhibition of autophagy suppresses H/R induced ferroptosis and cell injury

**Fig. 4:**
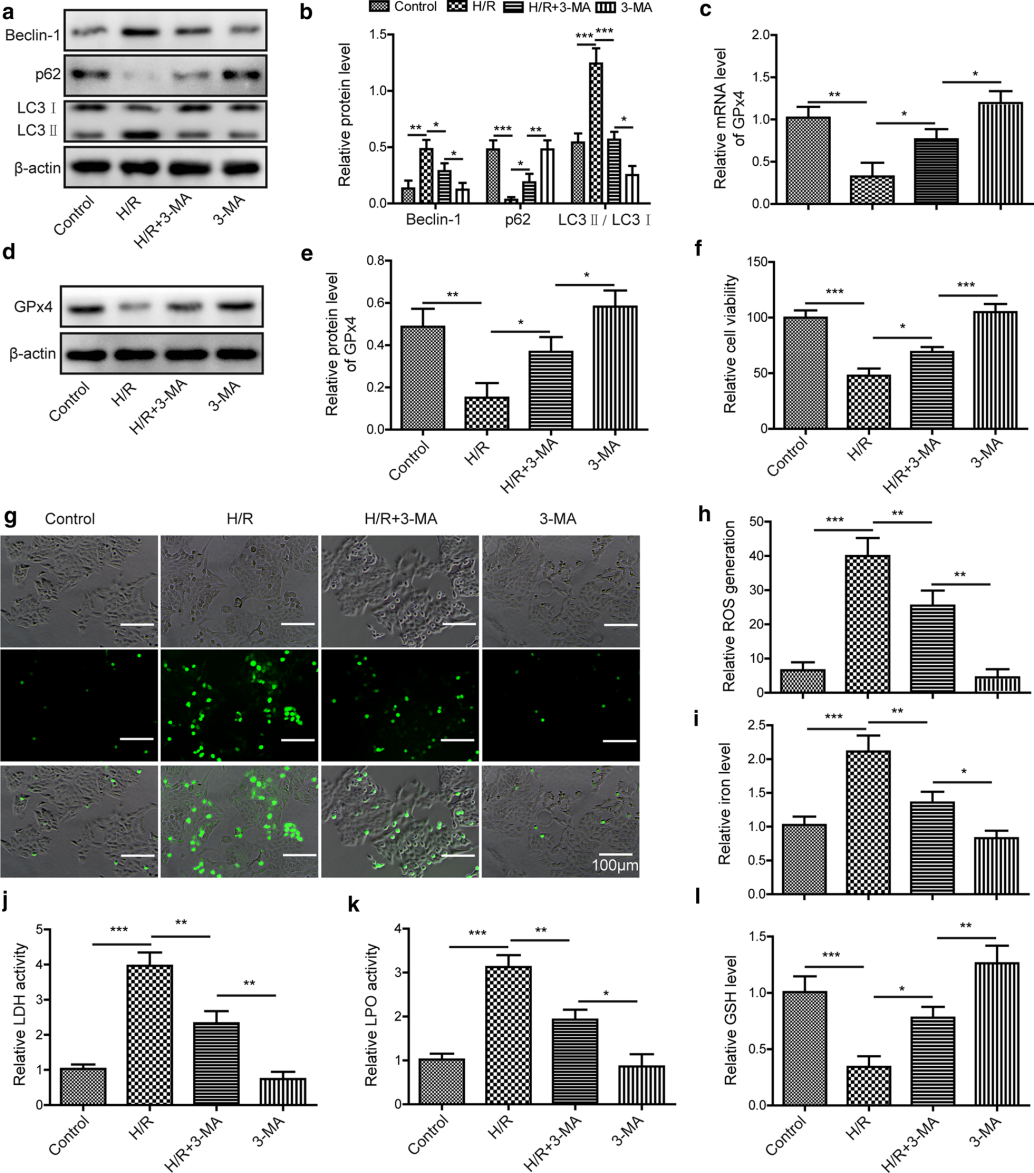
Inhibition of autophagy suppresses H/R induced ferroptosis and cell injury. Myocardial cells were pretreated with 3-MA (5 mM) for 24 h before being subjected to H/R. **a**, **b** Relative protein levels of Beclin-1, p62 and LC3 were determined by western blotting. **c**–**e** Relative mRNA and protein levels of GPx4 in cells were assessed respectively. **g** CCK8 assay was performed to assess cell viability. (G&H) Relative cellular ROS levels in 3-MA treated cells were measured after H/R. Relative cellular iron level (**i**), LDH activity (**j**), LPO level (**k**) and GSH level (**l**) in each group of cells were detected by corresponding assay kits after H/R. The results were presented as the mean ± SD. n = 3; *P < 0.05, **P < 0.01, ***P < 0.001

To directly test the relationship between autophagy and ferroptosis during myocardial I/R injury, we used the autophagy inhibitor, 3-methyladenine (3-MA), and assessed its effects on H/R induced ferroptosis and cell damage. As expected, 3-MA treatment significantly suppressed the increases of Beclin-1 and LC3 in protein caused by H/R, but recovered the levels of p62 (Fig. 4a, b), suggesting that H/R induced autophagy was restrained by 3-MA. The mRNA and protein levels of GPx4 in cells exposed to H/R were partially restored to the baseline by 3-MA (Fig. 4c–e). Further, 3-MA treatment recovered the reduced cell viability of H/R exposed cells (Fig. 4f), while H/R induced increase of cellular ROS level was suppressed by 3-MA treatment (Fig. 4g, h). Consistently, the changes of cellular iron, LPO, GSH levels, and LDH activity caused by H/R were all restored to baseline when cells were treated with 3-MA (Fig. 4i–l). Al-together, these data provide an evidence that inhibition of autophagy could suppress H/R-induced ferroptosis and myocardial cell injury.

### Knockdown of ELAVL1 suppresses I/R induced ferroptosis and injury via inhibiting autophagy

**Fig. 5:**
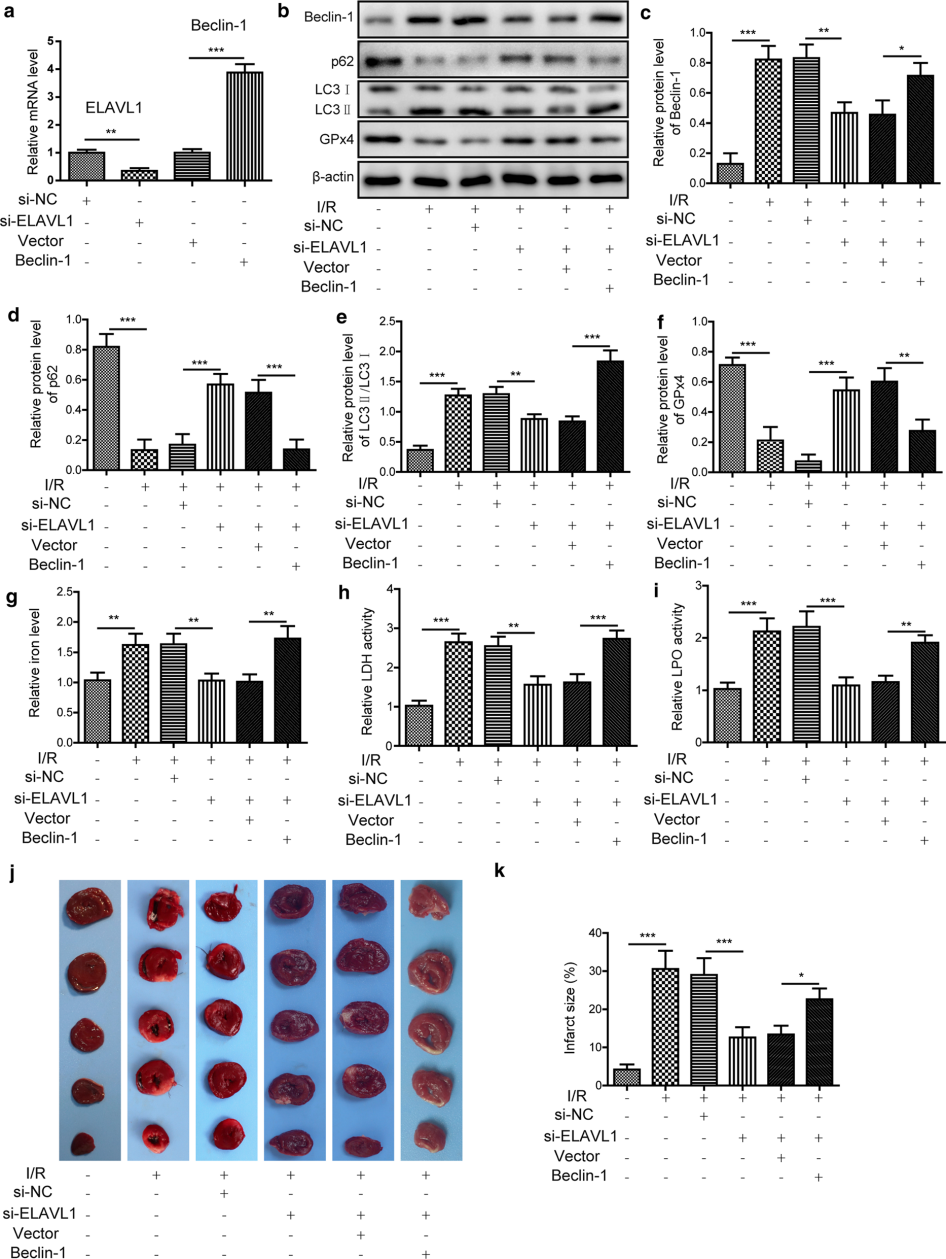
Knockdown of ELAVL1 suppresses I/R induced ferroptosis and injury via inhibiting autophagy. For inhibition of ELAVL1 or overexpression of Beclin-1, mice were received injection of lenti-si-ELAVL1 or Beclin-1. **a** Relative mRNA levels of ELAVL1 and Beclin-1 were measured by qRT-PCR. **b**–**f** Relative protein levels of p62, GPx4, Beclin-1, and LC3 were determined by western blotting. Relative iron level **g**, LDH activity **h** and LPO level **i** were assessed by corresponding assay kits. **j** Representative TTC staining images of myocardial sections from each group of mice. **k** Quantifications of infarct size in each group of mice. The results were presented as the mean ± SD. n = 5; *P < 0.05, **P < 0.01, ***P < 0.001

Then we investigated the mechanisms underlying ELAVL1 anti-ferroptosis during I/R, and we focused on the autophagy as our aforementioned results showed that ELAVL1 restrained autophagy as well. As expected, knockdown of ELAVL1 through siRNA reversed the changes of p62, GPx4, Beclin-1, and LC3 induced by I/R surgery via up-regulating p62 and GPx4 and down-regulating Beclin-1 and LC3 (Fig. 5a–f). However, co-overexpression of Beclin-1, which promoted autophagy, suppressed the effects of si-ELAVL1 (Fig. 5a–f). Similarly, the increases of cellular iron level, LDH activity and LPO level induced by I/R surgery were inhibited by si-ELAVL1 (Fig. 5g–i). Co-overexpression of Beclin-1 in si-ELAVL1-treated mice increased those levels again upon I/R surgery (Fig. 5g–i). Finally, knockdown of ELAVL1 via si-ELAVL1 significantly reduced the infarct size of myocardia (Fig. 5j, k). Nevertheless, overexpression of Beclin-1 together with si-ELAVL1 brought up the infarct size again (Fig. 5j, k). Taken together, these results prove that knockdown of ELAVL1 suppresses ferroptosis and ameliorates I/R injury through inhibiting autophagy.

### ELAVL1 directly binds and stabilizes Beclin-1 mRNA

**Fig. 6:**
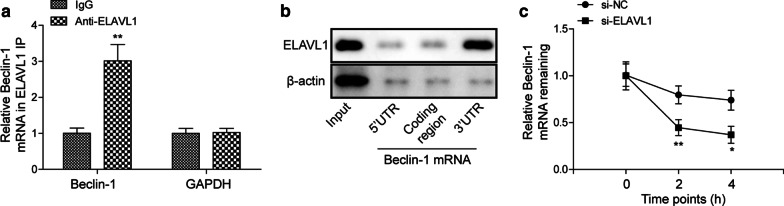
ELAVL1 directly binds and stabilizes Beclin-1 mRNA. **a** Relative Beclin-1 mRNA and GAPDH mRNA levels following ELAVL1 immunoprecipitation. **b** Relative ELAVL1 protein level following biotin pull-down. **c** Relative Beclin-1 mRNA levels in transfected cells following actinomycin D treatment. The results were presented as the mean ± SD. n = 3; *P < 0.05, **P < 0.01

Previous study suggests that ELAVL1 directly binds to Beclin-1 mRNA in hepatic stellate cells to regulate autophagy (Zhang 2018). We wondered whether similar interaction existed in myocardial cells. Using RIP assay, we found that ELAVL1 antibody pulled down significantly more Beclin-1 mRNA but not GAPDH mRNA compared to control IgG antibody (Fig. 6a), suggesting that ELAVL1 directly interacts with Beclin-1 mRNA in myocardial cells. We further characterized the binding region using biotinylated transcripts. As shown in Fig. 6b, we found that ELAVL1 bound with 3′UTR of Beclin-1 mRNA, but not 5′UTR or the coding region. We also examined how the binding affected the Beclin-1 mRNA stability. In control si-NC-transfected myocardial cells, application of the transcription inhibitor, actinomycin D, gradually diminished the Beclin-1 mRNA levels with time (Fig. 6c). However, in si-ELAVL1 transfected cell, the reduction in Beclin-1 mRNA induced by actinomycin D was consistently and significantly more than that in control cells (Fig. 6c). These findings indicate that ELVAL1 directly binds with and stabilize Beclin-1 mRNA.

### FOXC1 activates ELAVL1 during H/R

**Fig. 7:**
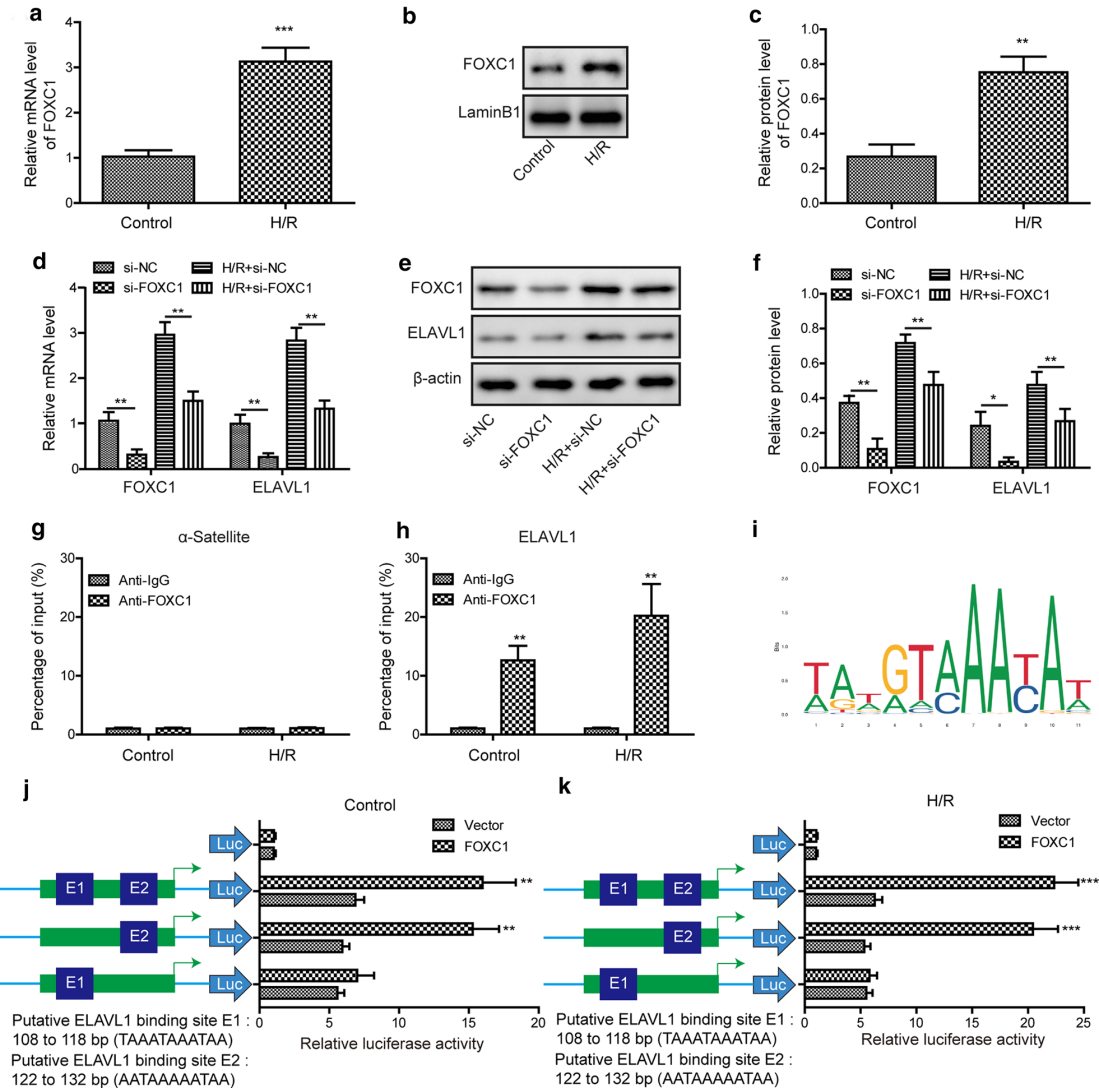
FOXC1 activates ELAVL1 during H/R. **a**–**c** Relative mRNA and protein levels of FOXC1 in cells upon control or H/R exposure. **d**–**f** Relative mRNA and protein levels of FOXC1 and ELAVL1 in si-FOXC1 transfected cells following H/R. **g** Relative α-Satellite level pulled down by FOXC1 antibody or control IgG antibody during control or H/R. **h** Relative ELAVL1 level pulled down by FOXC1 antibody or control IgG antibody during control or H/R. **i** FOXC1 binding motif provided by the JASPAR database. **j** Relative luciferase activities of empty vectors or vectors containing E1, E2, or both under baseline. **k** Relative luciferase activities of empty vectors or vectors containing E1, E2, or both following H/R exposure. The results were presented as the mean ± SD. n = 3; *P < 0.05, **P < 0.01, ***P < 0.001

FOXC1 is a transcription factor that has been implicated in myocardial I/R injury (Zhang 2019). To test the mechanisms underlying ELAVL1 elevation following I/R surgery, we tested whether FOXC1 regulated ELAVL1 transcription. Exposure myocardial cells to H/R increased FOXC1 mRNA level, as well as protein level (Fig. 7a–c). Transfection of cells with si-FOXC1 substantially diminished mRNA and protein levels of FOXC1 and suppressed the increase of FOXC1 expression induced by H/R exposure (Fig. 7d–f). Interestingly, we observed that knockdown of FOXC1 in control cells also decreased ELAVL1 level and that H/R induced upregulation of ELAVL1 level was reversed by FOXC1 knockdown as well (Fig. 7d–f), suggesting that FOXC1 regulates ELAVL1 expression. To further investigate that, we performed ChIP experiment. Immunoprecipitation with specific FOXC1 antibody successfully pulled down ELAVL1 promoter but not α-Satellite, suggesting that FOXC1 directly binds ELAVL1 promoter (Fig. 7g, h). Moreover, following H/R exposure, FOXC1 antibody pulled down more ELAVL1, indicating a stronger binding (Fig. 7g, h). We next examined which binding site mediated the regulation as we found two potential binding sites (E1 and E2) (Fig. 7i–k). Vectors containing the E1 binding site did not show any difference in luciferase activity compared to empty vector control while vectors containing the E2 binding site showed enhanced luciferase activity (Fig. 7j), suggesting that E2 is the functional binding site. Again, we observed a higher increase of luciferase activity following H/R exposure (Fig. 7k), confirming that H/R exposure enhances the binding. These results demonstrate that FOXC1 activates ELAVL1 transcription and H/R increases the transcription of ELAVL1 mediated by FOXC1.

## Discussion

Cardiovascular disease like coronary heart disease is the top cause of death in the world, especially in the Western world (Shi et al. 2016). As the primary type of coronary heart disease, myocardial infarction affects millions of people worldwide (Aguero 2015; Lu et al. 2015). Currently the treatment of myocardial ischemia is limited and the damages are usually irreversible. Here, we studied the role of ferroptosis and ELAVL1 in myocardial I/R to provide insights into the molecular mechanisms. We showed that ferroptosis was induced and ELAVL1 was elevated following myocardial I/R surgery. Knockdown of ELAVL1 suppressed ferroptosis and myocardial I/R injury. Besides, suppression of ELAVL1 inhibited autophagy and inhibition of autophagy restrained ferroptosis and myocardial I/R injury. Molecularly, we identified that ELAVL1 bound with Beclin-1 mRNA and stabilized it. FOXC1 bound to ELAVL1 promoter region and activated ELAVL14 transcription during myocardial I/R. Activation of autophagy reversed the effects of ELAVL1 knockdown on ferroptosis and myocardial I/R injury. Our study sheds light on the molecular mechanisms of myocardial I/R injury, providing avenues for the development of future therapeutic strategies.

Ferroptosis is a recently defined iron-dependent cell death (Mou 2019). Factors, such as suppressing system Xc- or GPx4, regulate glutathione peroxidase and decrease the antioxidant capacity, leading to accumulation of lipid ROS and eventually oxidative cell death (Li 2020; Lei et al. 2019). Ferroptosis has been implicated in pathological conditions including neurodegenerative diseases (Alzheimer’s and Parkinson’s diseases), carcinogenesis, and kidney degeneration (Li 2020; Han 2020). Recently some studies reported ferroptosis in I/R injury (Li et al. 2020; Kobayashi 2018), but the exact function is not clear. Here, we provided strong evidence that myocardial I/R surgery induced substantial ferroptosis in the myocyte. We observed ferroptosis-specific changes including increases of cellular iron level and ROS signaling accompanied by decreased GPx4 and GSH levels following myocardial I/R injury or H/R exposure. Further, we showed that ferroptosis played an active and important role in the myocardial I/R injury. Inhibition of ferroptosis tremendously reduced the injury. Therefore, ferroptosis could serve as a target for protection against myocardial I/R injury (Fang 2019).

ELAVL1 is a ubiquitous RBP and a key regulator of cytoplasmic mRNA fate (Chang and Hla 2014). Despite its primary location in nucleus, ELAVL1 translocates to cytoplasm to stabilize ARE-containing mRNAs and promote their expression upon cellular activation or stress conditions like oxidative stress and autophagy (Chang and Hla 2014). Numerous mRNAs have been shown to bind with ELAVL1, such as *BCL2* (apoptosis regulator), *HIF1A* (hypoxia inducible factor 1 subunit alpha), *TNF* (tumor necrosis factor), *NOS2* (nitric oxide synthase 2) (Chang 2014, 2013; Skliris 2015). Therefore, ELAVL1 is largely involved in many processes including apoptosis, autophagy, and oxidative stress (Srikantan and Gorospe 2012; Katsanou 2009; Zucal 2015). Here, during myocardial I/R injury wherein excessive ROS and inflammatory cytokines were produced (Zhou et al. 2015), we saw a substantial increase of ELAVL1. Further, this up-regulation plays a positive role in autophagy and ferroptosis in that knockdown of ELAVL1 greatly suppressed those processes, thus ameliorating I/R injury. Similar promoting role of ELAVL1 in ferroptosis has been reported during liver fibrosis (Zhang 2018), suggesting a conserved role of ELAVL1 in cell or tissue damage. Moreover, Beclin-1 mRNA has been shown as a target of ELAVL1 to trigger autophagy and ferroptosis in liver fibrosis (Zhang 2018). Consistently, inhibition of autophagy could suppress ferroptosis in H/R induced HCM cells and Beclin-1 could reversed the effects of ELAVL1 silencing on myocardial I/R injury in mice via regulating autophagy and ferroptosis. We also confirmed a direct interaction between ELAVL1 and Beclin-1 mRNA and showed that this binding promotes Beclin-1 mRNA stability, which could be the mechanism through which ELAVl1 accelerates autophagy process. In addition, it will be interesting to examine if there are other targets mediating ELAVL1′s role in autophagy and ferroptosis (Kang and Tang 2017).

The increase of ELAVL1 induced by myocardial I/R is mediated by FOXC1-activated transcription. FOXC1 is a transcription factor that plays an important role in heart and cardiovascular development (Lambers 2016; Kume 2009). Dysregulation of FOXC1 has been reported in diseases including congenital heart defects, cancers, and myocardial ischemia (Zhang 2019; Elian et al. 2018). FOXC1 expression could be induced by hypoxia and subsequently it can activate the expression of toll-like receptors, contributing to the inflammatory responses and cell injury (Zhang 2019). Here, we identified ELAVL1 as a novel target of FOXC1. FOXC1 directly bound to ELAVL1 promoter region and promotes ELAVL1 transcription. Furthermore, this interaction was enhanced following H/R exposure, resulting higher level of ELAVL1. Notably, previous studies have shown that ELAVL1 could target FOXC1 mRNA to promote its stability (Katsanou 2009). This implies that ELAVL1/FOXC1 might form a positive feedback loop during H/R. Our study, together with previous work, show that FOXC1 is a key player in the myocardial I/R injury.

## Conclusions

In summary, we showed that autophagy-dependent ferroptosis contributes to the myocardial I/R injury and that this ferroptosis is caused by ELAVL1, leading to overproduction of lipid signaling. Additionally, FOXC1 activates ELAVL1 transcription in myocardial I/R injury. Targeting ferroptosis or FOXC1/ ELAVL1 could be beneficial for myocardial ischemia patients.

## Data Availability

All data generated or analyzed during this study are included in this article. The datasets used and/or analyzed during the current study are available from the corresponding author on reasonable request.
