# Correction: ELAVL1 is transcriptionally activated by FOXC1 and promotes ferroptosis in myocardial ischemia/reperfusion injury by regulating autophagy

**DOI:** 10.1186/s10020-022-00529-x

**Published:** 2022-08-15

**Authors:** Hui-Yong Chen, Ze-Zhou Xiao, Xiao Ling, Rong-Ning Xu, Peng Zhu, Shao-Yi Zheng

**Affiliations:** 1 Department of Cardiovascular Surgery, Nanfang Hospital, Southern Medical University, No. 1838 North Guangzhou Avenue, Baiyun District, Guangzhou, 510515 Guangdong People’s Republic of China; 2 Department of Thoracic Surgery, Yuebei People’s Hospital, Shantou University, Shaoguan, 512026 Guangdong People’s Republic of China

## Correction: Mol Med (2021) 27:14 https://doi.org/10.1186/s10020-021-00271-w

Following publication of the original article (Chen et al. 2021), the authors informed us that there are some mistakes in the manuscript due to their carelessness. The image of H/R group in Fig. 2E and the image of H/R + 3-MA group in Fig. 4G was repeated.
